# Diversity and characterization of bacteria associated with the deep-sea hydrothermal vent crab *Austinograea* sp. comparing with those of two shallow-water crabs by 16S ribosomal DNA analysis

**DOI:** 10.1371/journal.pone.0187842

**Published:** 2017-11-09

**Authors:** Na Zhang, Chengwen Song, Minxiao Wang, Yuan Liu, Min Hui, Zhaoxia Cui

**Affiliations:** 1 Key Laboratory of Experimental Marine Biology, Institute of Oceanology, Chinese Academy of Sciences, Qingdao, China; 2 Key Laboratory of Marine Ecology and Environmental Sciences, Institute of Oceanology, Chinese Academy of Sciences, Qingdao, China; 3 Deep Sea Research Center, Institute of Oceanology, Chinese Academy of Sciences, Qingdao, China; 4 Laboratory for Marine Biology and Biotechnology, Qingdao National Laboratory for Marine Science and Technology, Qingdao, China; 5 National & Local Joint Engineering Laboratory of Ecological Mariculture, Qingdao, China; National Cheng Kung University, TAIWAN

## Abstract

For deep-sea hydrothermal vent crabs, recent investigations have revealed some epibiotic bacteria, but no study has described the bacterial community associated with the gill and intestine. In this study, the microbiota attached to the gill and intestine of the hydrothermal vent crab *Austinograea* sp. and two shallow-water crab species (*Eriocheir sinensis* and *Portunus trituberculatus*) were compared by high-throughput sequencing of 16S rDNA genes. The highest and lowest diversity in bacterial communities were observed in the gill and intestine of *Austinograea* sp., respectively. Non-metric multidimensional scaling (NMDS) analysis indicated that *Austinograea* sp. harbored a distinct microbial community. Operational taxonomic units (OTUs) for phylum *Fusobacteria*, class *Epsilonproteobacteria*, and genera *Leucothrix*, *Polaribacter*, *Fusibacter*, etc. were dominant in *Austinograea* sp. Of these, *Leucothrix*, *Sulfurospirillum*, and *Arcobacter* may be involved in oxidizing reduced sulfur compounds and sulfur metabolism; *Marinomonas*, *Polaribacter* adapted to the low temperature, and *Fusibacter* and *Psychrilyobacter* may survive well under hypoxic conditions. Bacteria commonly present in seawater were dominant in the gill, whereas anaerobic bacteria showed strikingly high abundance in the intestine. Interestingly, *Firmicutes* and *Epsilonproteobacteria* may complement each other in *Austinograea* sp., forming an internal environment. The diversified microbial community of *Austinograea* sp. reveals adaptation to the hydrothermal vent environment.

## Introduction

The deep-sea hydrothermal vent environment is one of the most variable ecosystems on Earth, and the fauna inhabiting this environment need special adaptations to tolerate the harsh conditions [1]. In such ecosystems, life is based on chemosynthetic primary production and widespread microbial-invertebrate associations [2]. The associations between hydrothermal vent invertebrates and chemosynthetic symbionts are diverse, including loose epibiosis and obligate endosymbiosis [2]. Decapod crustaceans usually exhibit a diverse range of bacterial associates such as chemoautotrophs and methanotrophs [3,4]. A number of studies suggest that some chemoautotrophic epibionts play a nutritional role in the hydrothermal shrimp, *Rimicaris exoculata* [5–8], and different microbial communities are hypothesized to participate in different inorganic metabolic pathways [9]. *Epsilonproteobacteria* also supply nutrition to the host by metabolizing sulfur compounds in some deep-sea hydrothermal vent invertebrates such as shrimps or crabs [3,4,9–16], and tubeworms [17].

In addition, invertebrate-associated bacteria in hydrothermal vents not only have host specificity, but also organ specificity within the host’s gut or gill chamber [10,11,18–23], consistent with their nutrient sources [4,24] and challenging hydrothermal vent environments [7,10]. Many studies have been conducted to explore the gill-associated microbiota in hydrothermal vent invertebrates. Hydrothermal vent mytilid mussels with vesicomyid clams and some provannid gastropods harbor symbiotic bacteria in their gills [18–20], and the hydrothermal shrimp, *R*. *exoculata*, also harbors epibiotic bacteria on the inner side of its enlarged gill chambers [21–23]. Besides the epibionts on the gill chamber, some microflora is gut specific and may be implicated in nutrition of hydrothermal vent shrimps [8,10,11,24]. Earlier observations have also elucidated the epibionts associated with the setae of deep-sea hydrothermal vent decapod [3,13–16,25,26]. In the crab *Xenograpsus testudinatus* inhabiting in shallow-water hydrothermal vents, the dominant bacteria also have both host and potential organ specificities, consistent with a potential trophic transfer between host and bacteria [27]. However, little is known about the microbial communities harbored in the gill and intestine of deep-sea hydrothermal vent crabs.

*Austinograea* sp., which belongs to the order Brachyura, family Bythograeidae, is the most common and abundant species and the top predator in hydrothermal vent [28]. All species have whitish, smooth and rounded carapaces, vestigial eye stalks and resemble each other in gross morphology [29]. Apart from the morphological description [30–33] and mitochondrial genomes [28,34], reports of these species are very limited and it is not clear how they adapt to the deep-sea hydrothermal vent environment. Transcriptomes of four tissues (eyestalk, gill, hepatopancreas and muscle) of *Austinograea alayseae* are performed and reveal the molecular basis of adaptive evolution [35], while no data are collected for the bacterial associations and their roles in crab adaptive evolution.

High-throughput sequencing (HTS) of 16S rDNA allows exploration of microbial diversity at an unprecedented scale [36]. Here, we described and characterized, for the first time, the composition of the microbial communities associated with the gill and intestine of the deep-sea crab *Austinograea* sp. by HTS of 16S rDNA genes. The Chinese mitten crab *Eriocheir sinensis* H. Milne Edwards, 1853 (Crustacea: Decapoda: Brachyura) is a fresh water crab species, however spawns and mates in saline water. The swimming crab *Portunus* *trituberculatus* Miers, 1876- (Crustacea: Decapoda: Brachyura), is a sea-dwelling crab inhabiting in the seafloor with sand or pebbles. These two shallow-water crab species, representing contrasting geochemical settings and depths to a certain extent, were used as comparisons to illustrate the diversity in the microbial communities harbored in the gill and intestine of *Austinograea* sp. The current study may present a good example for the interactions between microorganisms and invertebrate hosts in deep-sea hydrothermal vents.

## Materials and methods

### Ethics statement

The experiments were conducted in strict accordance with the guidelines of the Institutional Animal Care and Use Committee (IACUC) of the Chinese Academy of Sciences (No. 2011–2). This study was specifically approved by the Committee on the Ethics of Animal Experiments of the Institute of Oceanology at the Chinese Academy of Sciences. All efforts were made to minimize the suffering of the crabs.

### Sample collection

Healthy Chinese mitten crabs (*E*. *sinensis*) and swimming crabs (*P*. *trituberculatus*) were collected from Liaohe River of Liaoning province and Qingdao coast of Shandong province, respectively. Deep-sea hydrothermal vent crabs were collected between two dive sites (#30/Desmos and #32/Pacmas, Table 1) in the South China Sea by the remotely operated vehicle (ROV) *Faxian*, which was deployed using the RV *KEXUE*. Desmos and Pacmas are the two fields in the Manus Basin, where low pH and high concentrations of H_2_S and SO_4_^2-^ were detected [37]. Other environment data and microbial communities in the sediments of these two sites have been published by Wang et al. [38]. Fresh intestines and gills of *E*. *sinensis* (72 ± 0.2 g) and *P*. *trituberculatus* (135 ± 0.2 g) were collected from four live individuals for genomic DNA extraction. Samples from *Austinograea* sp. were kept at -80°C before used for genetic analysis. Specifically, half of the gills (the other half was used for the transcriptome sequencing) and the separated entire intestine in each crab were dissected for further analyses and also four individuals (5.5 ± 0.1 g) were treated (S1 Fig).

**Table 1:** Details of the dive sites where deep-sea crabs were collected.

Location	Sampling date	Coordinates	Depth	Salinity	Temperature
Desmos	2015.6.9	151°52′50.084″E, 3°42′47.259″S	1995 m	35.67‰	1.01°C
Pacmas	2015.6.11	151°40′09.137″E, 3°43′41.246″S	1680 m	35.67‰	1.15°C

### Genomic DNA extraction and PCR amplification

Total DNA from the gills and intestines were extracted by the phenol-chloroform method as described by Sambrook and Russell [39]. The concentration of DNA samples were diluted to 1 ng/μL using sterile water after measured by a spectrophotometer and their purity were determined by 1% agarose gel electrophoresis.

The 16S rDNA genes of hypervariable regions 16SV3-V4 were amplified using the following specific primers with the barcode: 341F (CCTAYGGGRBGCASCAG) and 806R (GGACTACNNGGGTATCTAAT). Barcodes added to the specific primer for distinguishing different samples were shown in S1 Table. PCR was performed in 30 μL volumes containing 15 μL of Phusion^®^ High-Fidelity PCR Master Mix (New England Biolabs), 0.2 μmol of forward and reverse primers, and about 10 ng template DNA. Thermal cycling consisted of initial denaturation at 98°C for 1 min, followed by 30 cycles of denaturation at 98°C for 10 s, annealing at 50°C for 30 s, and elongation at 72°C for 30 s, and a final elongation of 5 min at 72°C. PCR products mixed with same volume of 6 × loading buffer (contained SYB green) were examined on 2% agarose gel. Samples with a bright main band between 400–450 bp were chosen for further experiments.

### HTS of bacterial 16S rDNA

PCR products mixed in equidensity ratios were purified using the Qiagen Gel Extraction Kit (Qiagen, Germany). Sequencing libraries were generated using TruSeq^®^ DNA PCR-Free Sample Preparation Kit (Illumina, USA) following the manufacturer's recommendations and index codes were added. The samples were then sequenced on an Illumina HiSeq2500 platform.

### Statistical and bioinformatics analysis

Paired-end reads, merged using FLASH (V1.2.7, http://ccb.jhu.edu/software/FLASH/) [40], were assigned to samples based on their unique barcode. Quality filtering on the raw tags was performed under specific filtering conditions to obtain high-quality clean tags [41] according to the QIIME (V1.7.0, http://qiime.org/index.html) [42] quality controlled process. The effective tags were obtained by comparison with the reference database (Gold database, http://drive5.com/uchime/uchime_download.html) and removing the chimera sequences.

Sequences analyzed by Uparse software (Uparse v7.0.1001, http://drive5.com/uparse/) [43] with similarity not less than 97%, were assigned to the same Operational Taxonomic Units (OTUs). The representative sequence for each OTU was annotated by the RDP classifier (Version 2.2, http://sourceforge.net/projects/rdp-classifier/) algorithm to obtain taxonomic information [44]. Alpha diversity was applied in analyzing the complexity of species diversity for a sample through 6 indices, including Observed-species, Chao1, Shannon, Simpson, ACE, and Good’s-coverage. All these indices in our analyses were calculated with QIIME (Version 1.7.0) and displayed with R software (Version 2.15.3). The indices of Chao1 and ACE estimator were selected to identify community richness. Shannon and Simpson indices were used to identify community diversity whereas Good’s coverage was calculated to characterize the sequencing depth. The significant levels between all samples were detected by T-test, Wilcox-test and Tukey-test, and *P* < 0.05 meant significant different between two samples. Non-metric multidimensional scaling (NMDS) based on weighted UniFrac distance was shown to give a visual comparison of the pairwise UniFrac distance among samples. Phylogenetic analysis was conducted to exhibit the distribution of major microbial communities in different groups by using the software MUSCLE (Version 3.8.31, http://www.drive5.com/muscle/) [45]. The linear discriminant analysis (LDA) effect size (LEfSe) method (http://huttenhower.sph.harvard.edu/lefse/) [46] was performed to characterize the specialization of microorganisms from different groups. All group data were calculated by the average of four specimens from each species.

## Results

### Microbial relative abundance and diversity

In total, 227,785 (111,974 for gills and 115,811 for intestines) effective tags were obtained from *Austinograea* sp., *E*. *sinensis*, and *P*. *trituberculatus* by HTS. By the species associated bacterial composition analysis, 1,118 and 1,050 OTUs were collected from the gill and intestine of *Austinograea* sp., respectively, 1,309 and 967 OTUs were collected from *E*. *sinensis*, whereas 1,114 and 1,657 OTUs were obtained from *P*. *trituberculatus*, respectively (Table 2). The diversity indices, including the Shannon, Simpson, and community richness- Chao1 and ACE all revealed microbiota variations between the *Austinograea* sp. and two shallow-water crab species, and even between the gill and intestine from *Austinograea* sp. In all three crabs, the highest microbial diversity was observed in the gill of the hydrothermal vent crab (5.18 in Shannon index), and the lowest bacterial diversity was found in intestine of *Austinograea* sp. (3.87 in Shannon index).

**Table 2:** Similarity-based OTUs and species richness of the bacterial phylotypes in gills and intestines of three species.

Sample name	Effective tags	OTU	Shannon	Simpson	Chao1	ACE
AG	37,199	1,118	5.18	0.90	763.53	787.64
EG	38,922	1,309	4.75	0.88	933.93	983.70
PG	35,854	1,114	4.73	0.82	741.11	790.25
AI	37,231	1,050	3.87	0.80	691.11	760.20
EI	36,589	967	4.48	0.87	619.89	660.45
PI	41,991	1,657	4.44	0.85	1210.51	1242.40

**Abbreviations**: for sample names, the first letter represents species- A for *Austinograea* sp., E for *E*. *sinensis*, P for *P*. *trituberculatus*; the second letter represents tissues- G for gill, I for intestine.

### Composition of microbiota in the gills and intestines from three species of crabs

**Fig 1:**
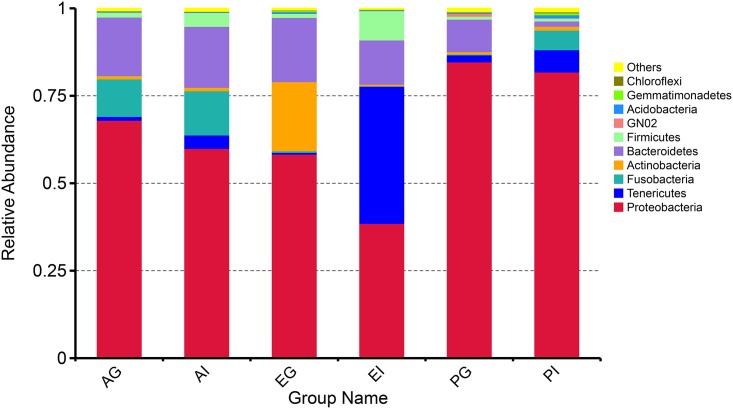
Relative abundance of predominant bacterial communities in the gills and intestines of *Austinograea* sp. and the other two shallow-water crab species at the phylum level. A color-coded bar plot shows the average bacterial genus distribution in different samples.

Based on the annotated information of the microbial 16S rDNA data obtained by HTS, 39 different phyla of bacteria were observed in the gills and intestines of three crab species. The top 10 phyla accounting for more than 99% of the microbial communities in each sample were selected to represent the relative abundance of various microbiota in different species (Fig 1). In the three crabs, *Proteobacteria* was the most predominant phylum in the gills and intestines of all specimens (68.0% in *Austinograea* sp., 58.4% in *E*. *sinensis*, and 84.7% in *P*. *trituberculatus*, respectively). In addition, the phyla *Bacteroidetes*, *Tenericutes* and *Firmicutes* were also found to be predominant microbes in the gills and intestines of the three crab species. Interestingly, a higher abundance of phylum *Fusobacteria* was observed in the gill and intestine of *Austinograea* sp. than in the other two crabs.

**Fig 2:**
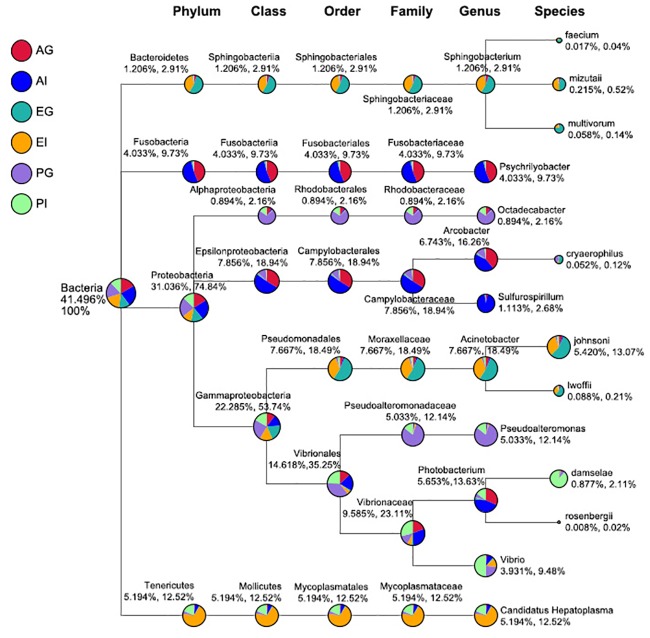
The taxonomic tree for bacteria in the gills and intestines of the three crab species. The sector with different colors represents different samples. The size of the sector indicates the relative abundance of a sample in this taxon. The two digits under the taxon indicate the relative abundance percentage in all taxa and the selected taxon, respectively.

At the genus level, four predominant phyla could be assigned into ten different genera (Fig 2). In *Austinograea* sp., *Psychrilyobacter* (*Fusobacteria*), *Arcobacter*, and *Photobacterium* (*Proteobacteria*) were the dominant genera both in the gill and intestine, and *Sulfurospirillum* was predominant in the intestine. In *E*. *sinensis*, *Sphingobacterium* (*Bacteroidetes*) and *Acinetobacter* (*Proteobacteria*) were the dominant genera both in the gill and intestine, and *Candidatus Hepatoplasma* (*Tenericutes*) was predominant in the intestine. The most abundant sequences in *P*. *trituberculatus* were affiliated to *Octadecabacter*, *Pseudoalteromonas*, and *Vibrio* (*Proteobacteria*).

### Differentiation of the microbiota in the gills and intestines between *Austinograea* sp. and two shallow-water crabs

**Fig 3:**
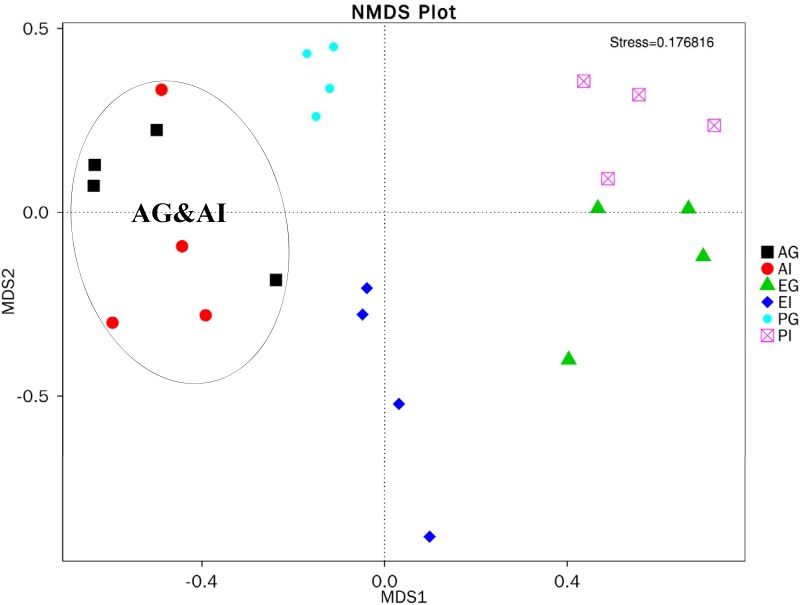
NMDS plot showing microbial community differences between the hydrothermal vent crab *Austinograea* sp. and the other two shallow-water crabs. Each point represents a sample. The distance between points indicates the degree of difference. The significant levels between different samples are evaluated by Tukey-test. Sample of the same group are shown in the same color. Stress < 0.2 indicates the reliability of the NMDS analysis.

An NMDS plot was generated to compare the relationship of the bacterial communities in the gills and intestines between *Austinograea* sp. and two shallow-water crabs at the OTU level. Microbial communities in the gill and intestine from *Austinograea* sp. obviously separated from those in the other two crabs as shown in Fig 3 (*P* < 0.05). This means that samples from the hydrothermal vent possess bacterial communities different from those in the samples from the shallow water.

**Fig 4:**
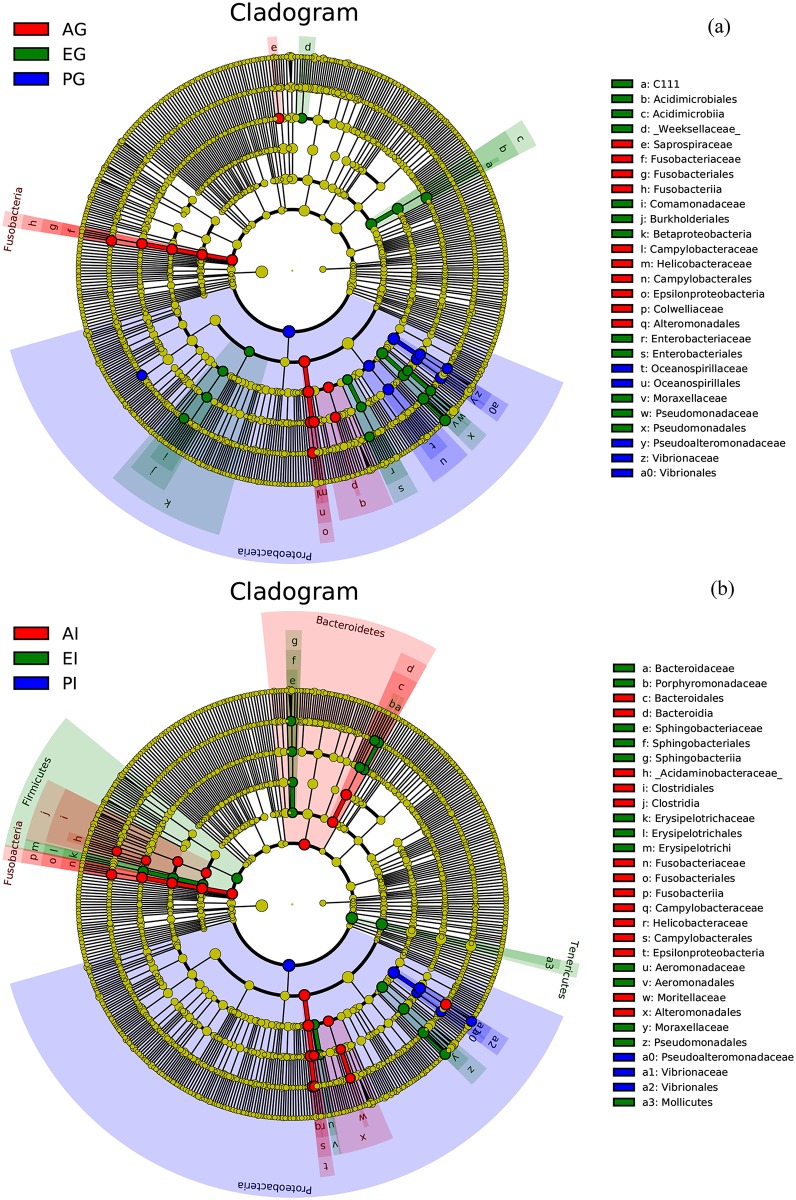
LEfSe identified the most differentially abundant taxa in the gills (a) and intestines (b) of the three crab species. Red, green, and blue colors represent the bacterial communities in the gills or intestines of *P*. *trituberculatus*, *E*. *sinensis*, and *Austinograea* sp., respectively. The dots on the cladogram represent the specific bacterial taxa whose colors are the same as those of the corresponding groups. The size of dots is proportional to the relative abundance.

To understand the differences among the bacterial groups in the gill and intestine from the three species of crabs, LEfSe was used to perform the comparisons among three crab species. The cladograms exhibited the structure of the microbiota in the gills and intestines of different species and their predominant bacteria (Fig 4). The greatest differences in taxa between the gills (Fig 4a) and intestines (Fig 4b) from different species were displayed. Thirteen specific microbial taxa at different levels were discovered in the gill of *Austinograea* sp. (Fig 4a). The first dominant taxon was the phylum *Proteobacteria*, especially the class *Epsilonproteobacteria*. The second specific taxon was the phylum *Fusobacteria*. Twenty-two unique bacterial categories were found in the intestine of the hydrothermal vent crab (Fig 4b). Similar to the gill, *Epsilonproteobacteria* was also a specific class in the intestine of *Austinograea* sp. Meanwhile, some other bacterial taxa jointly constituted the unique microbial communities in the intestine of *Austinograea* sp., including the phyla *Bacteroidetes* and *Fusobacteria*. The phylum *Firmicutes* were also predominant in the intestine of the hydrothermal vent crab.

**Fig 5:**
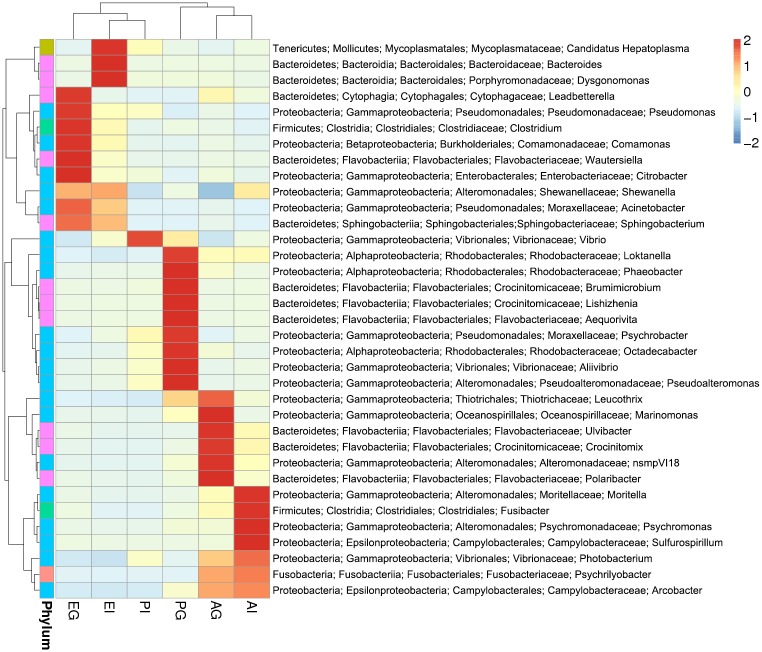
Heatmap of the microbial composition in the gills and intestines of the three crab species at the genus level. The heatmap indicates the relative abundance of each genus in different samples. The Y-axis clustering exhibits the variables and phylogenetic relationships of each bacterial species in different samples and the X-axis clustering indicates the phylogenetic relationships of these samples. The color intensity in the square grid represents the bacterial relative abundance, which is named as the z-value and generated by the relative abundance of bacterial species in each line after normalization treatment.

To further identify the specific bacterial taxa associated with the gill and intestine of hydrothermal vent crab clearly, a hierarchically clustered heatmap was selected to get an overview of the identified connections among the studied samples. Based on the bacterial 16S rDNA data and the microbial community abundance, the top 35 genera of bacteria from the gill and intestine of each species were used to construct the phylogenetic heatmap (Fig 5). Compared to the two shallow-water crabs, *Austinograea* sp. showed a higher abundance of the genera *Leucothrix*, *Marinomonas*, *Ulvibacter*, *Crocinitomix*, *nsmpVl18*, and *Polaribacter* in the gill; *Moritella*, *Fusibacter*, *Psychromonas*, and *Sulfurospirillum* in the intestine; and *Photobacterium*, *Psychrilyobacter*, and *Arcobacter* in both tissues.

## Discussion

The deep-sea hydrothermal vents are characterized by low oxygen, oligotrophic, high thermal gradient (2–350°C), and presence of various toxic chemical compounds, such as hydrogen sulfide (H_2_S), methane (CH_4_), and heavy metals [47,48]. The symbiotic microbes associated with the hydrothermal vent animals may be different from those in some shallow-water animals to support their hosts adaption to the extreme environment. In this study, we observed the diversity in bacterial communities associated with *Austinograea* sp. and compared it to that of shallow-water crabs, *P*. *trituberculatus* and *E*. *sinensis*. This is the first study to investigate the difference in the microbial composition and characteristics between deep-sea and shallow-water crabs.

### Phylogenetic diversity and constitutive characteristics of the microbiota in the gill and intestine of *Austinograea* sp.

The variation in microbial communities in the gills and intestines between *Austinograea* sp. and two shallow-water crabs might be affected by their different habitat environments. The surroundings of *P*. *trituberculatus* and *E*. *sinensis* are moderate, while the habitat of *Austinograea* sp. is one of the most extreme environments on Earth, where bacterial diversity (7.20–8.27 for Shannon) may be much greater than other marine microbial diversity [38,49]. The highest diversity of microbial communities observed in the gill of *Austinograea* sp. may also be relevant to these challenging conditions [37]. Inconsistent with the gills, the intestine of *Austinograea* sp. had the lowest diversity among these three crab species. This might be due to the fact that the intestine is relatively independent from the external environment. On the other hand, hydrothermal vents are oligotrophic ecosystems [50]. For food sources apart from the particulate matter falling from above and drifting of food materials from the surrounding regions, *Austinograea* sp. might be mainly dependent on harbour endosymbiotic chemosynthetic bacteria for nutrition [2]. In addition, the microbial diversity in gill and intestine of *Austinograea* sp. were higher than that of shallow-water hydrothermal vent crabs [27]. These results indicate that environment and food sources influence the composition of the gill and intestinal microbiota.

### Symbionts associated with sulfur metabolism in *Austinograea* sp.

Almost all vent-endemic animals are considered to have a close relationship with the endo- and/or epi- symbiotic chemoautotrophic microorganisms as the primary producers [51]. Chemoautotrophic *Epsilonproteobacteria* and *Gammaproteobacteria* are the predominant primary producers in both free-living and symbiotic microbial communities in global deep-sea hydrothermal fields [52,53]. *Epsilonproteobacteria* use sulfur compounds as both electron-donors and acceptors, and extend the energetically feasible habitats with versatile energy metabolisms. In this research, *Epsilonproteobacteria*, possibly involved in the oxidation-reduction of sulfur compounds and carbon fixation [9], were considered as a significant microbiota in the gill and intestine of *Austinograea* sp. unlike that in the shallow-water crabs. This finding agrees with the result of the comparison of genes participating in sulfur metabolism among the three crabs [35]. In the hydrothermal vent of the Manus Basin, *Epsilonproteobacteria* are the predominant bacterial class in the sediments [37] and gastropods [54]. Moreover, *Epsilonproteobacteria* are also found in the shallow-water hydrothermal vent crabs and sea water [27]. These results confirm that *Epsilonproteobacteria* is a common bacterial group in the hydrothermal systems [55].

The class *Epsilonproteobacteria* encompasses a single order *Campylobacterales*, which includes two families [56]. The genera *Campylobacter*, *Arcobacter*, *Sulfurospirillum*, and *Thiovulum* belong to the family *Campylobacteraceae*, whereas the genera *Helicobacter* and *Wolinella* form the family *Helicobacteraceae*. All the above-mentioned genera, except for *Thiovulum* and *Wolinella*, were found in the gill and/or intestine of *Austinograea* sp., suggesting a close relationship between these microbes and the habitat of *Austinograea* sp. Particularly, the genus *Sulfurospirillum* found in the intestine of *Austinograea* sp. with a very high abundance is the only organohalide-respiring *Epsilonproteobacteria* described so far. They can thrive in polluted habitats that include many toxic compounds [57]. Another bacterium involved in sulfur metabolism and inhabiting the gill and intestine of deep-sea crab was the genus *Arcobacter*, which is a novel sulfur-oxidizing *Epsilonproteobacteria* and an important member of the microbial communities at deep-sea vents [58]. The presence of these bacteria reflects the adaptation of the hydrothermal vent crab to extreme environments by associated microorganisms.

As a sulfur-oxidizer [12,59], the genus *Leucothrix* belonging to the class *Gammaproteobacteria* was observed to show higher abundance in the gill of *Austinograea* sp. in our study. This result indicates that the *Leucothrix* associated with *Austinograea* sp. may use reduced sulfur compounds as an energy source for their chemoheterotrophic growth and provide the host with nutrition [12,59]. *Epsilonproteobacteria*, as well as some *Gammaproteobacteria* form a significant microbiota in the gill and intestine of *Austinograea* sp., which is consistent with the discovery of gastropods in the hydrothermal vent of the Manus Basin [54]. These microorganisms are mainly involved in metabolism of sulfur or sulfur compounds. Otherwise, transcriptome analyses of these three crab species demonstrated a significantly higher amount of genes participating in sulfur metabolism in the *Austinograea* sp. than in the two shallow-water crab species [35]. All these discoveries reveal that sulfur and sulfur compounds should be the primary inorganic chemicals in the habitat of *Austinograea* sp.

### Symbionts associated with cold-adaptation in *Austinograea* sp.

Besides the characteristics of chemoautotrophs, some deep-sea *Gammaproteobacteria* are psychrophilic [60]. Some psychrophilic bacteria dominant in the deep-sea crab *Austinograea* sp., were identified as *Gammaproteobacteria*, including the genera *Marinomonas*, *Moritella*, and *Psychromonas*. The genus *Marinomonas* comprises the Gram-negative bacterial strains distributed in different marine environments and even in cold environments, such as deep-sea sediments [58,61]. Some of these strains have proven to be an excellent source of metabolic enzymes, which are involved in carbohydrate metabolism, oxidation, desulfation, serine protease-producing and so on [62–66]. In this study, a higher abundance of the genus *Marinomonas* was observed in the gill of *Austinograea* sp., which suggests that gill surface-associated *Marinomonas* might be of interest for the production of enzymes for host metabolism [23].

The genus *Moritella* has been known to consist solely of psychrophilic species and has been studied as model microorganisms for low-temperature-adapted enzymes and piezophilic adaptation of marine bacteria to the deep sea [67]. Since *Moritella* species are also facultative anaerobes, *Moritella* strains were identified from the intestines of hydrothermal vent crab as expected [10]. The genus *Psychromonas* includes piezophilic, halophilic, and psychrophilic species that are widely distributed in aquatic environments and are an important component of polar and deep-sea microbiota [68–71]. They are chemoorganotrophs, and so, *Austinograea* sp. might obtain a significant portion of their energy from bacterial symbionts, making the symbiosis a true mutualism.

Members of the diverse bacterial phylum *Bacteroidetes* have colonized virtually all types of habitats on earth. According to Bergey's Manual of Systematic Bacteriology [72], *Bacteroidetes* comprises four classes: *Bacteroidia*, *Flavobacteria*, *Sphingobacteria*, and *Cytophagia*. Among these, the *Bacteroidia* is a normal component of the microbiota in animals especially in the gastrointestinal tract (GIT) [73], and the other three classes belong to the environmental *Bacteroidetes*. Interestingly, three genera *Ulvibacter*, *Polaribacter*, and *Crocinitomix* (*Flavobacteria*) were mainly discovered in the gill of the deep-sea hydrothermal vents crab *Austinograea* sp., and then in the intestine. As they are well known degraders of polymeric organic matter [72], these gill and intestinal bacteria could also help the host to gain energy from otherwise refractory carbohydrate sources, as symbionts.

### Symbionts associated with hypoxia adaptation in *Austinograea* sp.

The deep sea is low of oxygen (O_2_) and the hydrothermal-vent crab itself has a specific adaptive mechanism for this extreme environment [1]. In addition, they are also dependent on symbiotic microorganisms to survive in hypoxic conditions. The phylum *Fusobacteria* contains facultative aerobic to obligate anaerobic organisms that ferment carbohydrates or amino acids and peptides to produce various organic acids [74]. These species occur in anoxic environments including sediments as well as in the oral or intestinal habitats of animals. The genus *Psychrilyobacter* belonging to the family *Fusobacteriaceae*, was a specific microbiota with a higher abundance in the gill and intestine of the deep-sea hydrothermal vent crab *Austinograea* sp. They can utilize sugars, amino acids and peptone as carbon sources and produce H_2_ and acetate as the major fermentation products [75].

The phylum *Firmicutes* is phenotypically, physiologically, and ecologically diverse and consists of at least 26 families and 223 genera [76]. The genus *Fusibacter* belonging to the family *Clostridiales*, is also a strictly anaerobic, thiosulfate-reducing bacterium and utilizes a limited number of carbohydrates to produce acetate, butyrate, CO_2_, and H_2_ as the end products from glucose fermentation [77]. In this study, a higher abundance of these bacteria in the deep-sea hydrothermal vent crab gill and intestine indicates that *Austinograea* sp. may be depend on these associated microorganisms for their metabolism to adapt to the anaerobic conditions in hydrothermal vents. Meanwhile, they can also use thiosulfate and sulfur as electron acceptors during glucose fermentation, with production of H_2_S [76]. Therefore, they complement each other with the *Epsilonproteobacteria* in *Austinograea* sp., forming an inner environment, allowing the crabs to adapt better to the extreme environment.

## Conclusions

In conclusion, aquatic animals have a persistent, close relationship with the surroundings that they inhabit. In our study, the microbiota of the deep-sea hydrothermal vents crab, *Austinograea* sp., show differences from those of two shallow-water crabs suggesting the impact of the environment and food source on the deep-sea hydrothermal vents crab. Moreover, different bacterial abundances between the gill and intestine of *Austinograea* sp. indicate the existence of tissue-specific bacteria, and the specific association of bacteria with the host.

## Supporting information

S1 Fig *Austinograea* sp. from -80°C frozen samples. (JPG)

S1 Table Barcodes added to specific primers in different samples. (DOCX)
