# Supplementary figures and images for: Diversity and characterization of bacteria associated with the deep-sea hydrothermal vent crab Austinograea sp. comparing with those of two shallow-water crabs by 16S ribosomal DNA analysis
Source: PLoS One. 2017 Nov 9;12(11):e0187842. doi: 10.1371/journal.pone.0187842 (PMC5679544; doi:10.1371/journal.pone.0187842)

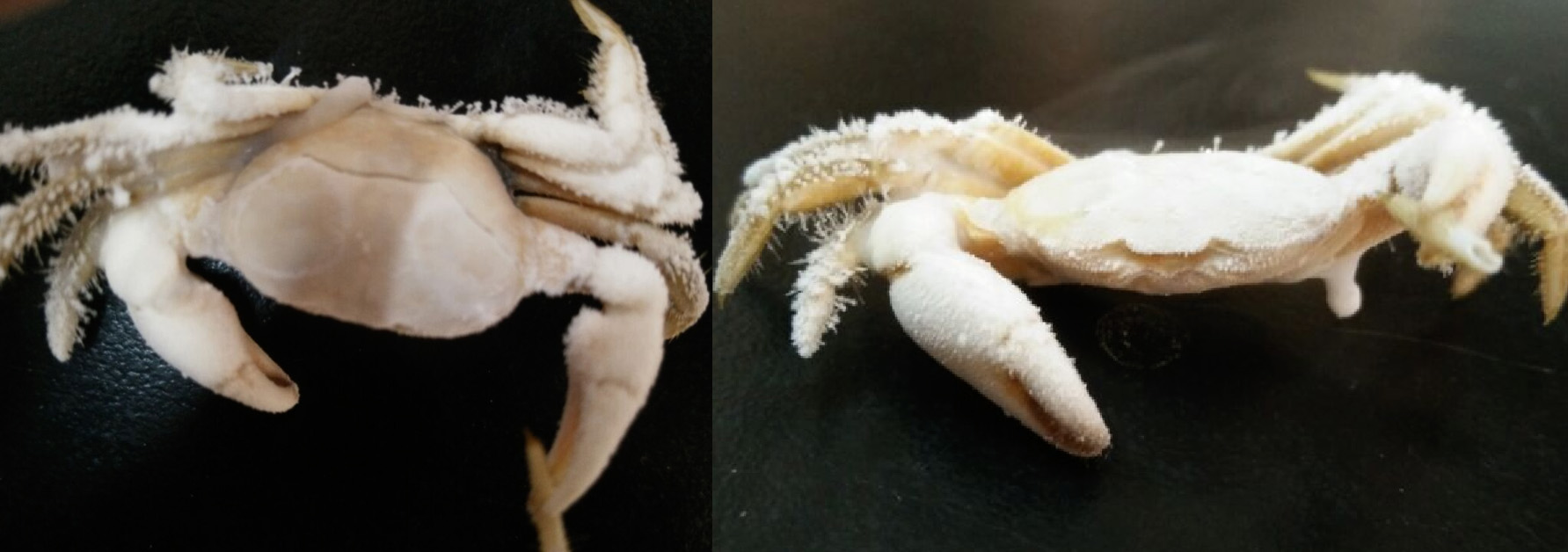

Supplement: S1 Fig — (JPG) [file pone.0187842.s001.jpg]
