# Supplementary material for: Diversity and characterization of bacteria associated with the deep-sea hydrothermal vent crab Austinograea sp. comparing with those of two shallow-water crabs by 16S ribosomal DNA analysis
Source: PLoS One. 2017 Nov 9;12(11):e0187842. doi: 10.1371/journal.pone.0187842 (PMC5679544; doi:10.1371/journal.pone.0187842)
Supplement: S1 Table — (DOCX) [file pone.0187842.s002.docx]

| Sample ID | Barcode Sequence |
| --- | --- |
| AG1 | ATCACG,CGTACG |
| AG2 | CGATGT,CGTACG |
| AG3 | TTAGGC,CGTACG |
| AG4 | TGACCA,CGTACG |
| AI1 | ACAGTG,CGTACG |
| AI2 | GCCAAT,CGTACG |
| AI3 | CAGATC,CGTACG |
| AI4 | ACTTGA,CGTACG |
| EG1 | GATCAG,CGTACG |
| EG2 | TAGCTT,CGTACG |
| EG3 | GGCTAC,CGTACG |
| EG4 | CTTGTA,CGTACG |
| EI1 | ATCACG,GGTAGC |
| EI2 | CGATGT,GGTAGC |
| EI3 | TTAGGC,GGTAGC |
| EI4 | TGACCA,GGTAGC |
| PG1 | ACAGTG,GGTAGC |
| PG2 | GCCAAT,GGTAGC |
| PG3 | CAGATC,GGTAGC |
| PG4 | ACTTGA,GGTAGC |
| PI1 | GATCAG,GGTAGC |
| PI2 | TAGCTT,GGTAGC |
| PI5 | ATCACG,ACTGAT |
| PI6 | CGATGT,ACTGAT |

**S1 Table Barcodes added to specific primers in different samples**
